# GSK-3 directly regulates phospho-4EBP1 in renal cell carcinoma cell-line: an intrinsic subcellular mechanism for resistance to mTORC1 inhibition

**DOI:** 10.1186/s12885-016-2418-7

**Published:** 2016-07-07

**Authors:** Hiromi Ito, Osamu Ichiyanagi, Sei Naito, Vladimir N. Bilim, Yoshihiko Tomita, Tomoyuki Kato, Akira Nagaoka, Norihiko Tsuchiya

**Affiliations:** Department of Urology, Yamagata University Faculty of Medicine, 2-2-2 Iida-Nishi, Yamagata, 990-9585 Japan; Division of Urology, Department of Regenerative and Transplant Medicine, Niigata Graduate School of Medical and Dental Sciences, 1-757, Asahimachi-dori, Chuo-ku, Niigata 951-8510 Japan

**Keywords:** Akt, GSK-3β, mTORC1, 4EBP1, Renal cell carcinoma, Rapamycin resistance, Combination index

## Abstract

**Background:**

The phosphoinositide 3-kinase (PI3K)/Akt/mammalian target of rapamycin 1 (mTORC1) signaling pathway is aberrantly activated in renal cell carcinoma (RCC). We previously demonstrated glycogen synthase kinase-3β (GSK-3β) positively regulated RCC proliferation. The aim of this study was to evaluate the role of GSK-3 in the PI3K/Akt/mTORC1 pathway and regulation of the downstream substrates, eukaryotic translation initiation factor 4E-binding protein 1 (4EBP1), ribosomal protein S6 kinase (S6K), and ribosomal protein S6 (S6RP).

**Methods:**

We used human RCC cell lines (ACHN, Caki1, and A498) and, as normal controls, human renal proximal tubular epithelial cell (HRPTEpC) and non-tumorous kidney tissues that were obtained surgically for treatment of RCC patients. Rapamycin-resistant ACHN (ACHN/RR) cells were generated with chronic exposure of ACHN to rapamycin ranging from 1nM finally to 1 μM. Cell viability, cell cycling and direct interaction between GSK-3β and 4EBP1 were evaluated with MTS assay, flowcytometry and in vitro kinase assay with recombinant GSK-3β and 4EBP1products, respectively. Protein expression and phosphorylation of molecules associated with the PI3K/Akt/mTORC1 pathway were examined by immunoblotting. Effects of drug combination were determined as the combination index with CompuSyn software.

**Results:**

Overexpression and phosphorylation of 4EBP1 and S6RP together with GSK-3 activation were observed in RCC cell lines, but not in human normal kidney cells and tissues. Cell proliferation, p4EBP1 and pS6RP were strongly suppressed by GSK-3 inhibition. Rapamycin and LY294002 sufficiently decreased pS6RP, but only moderately p4EBP1. In vitro kinase assays showed that recombinant GSK-3β phosphorylated recombinant 4EBP1, and the effect was blocked by GSK-3 inhibitors. Different from rapamycin, AR- A014418 remarkably inhibited cell proliferation, and rapidly suppressed p4EBP1 and pS6RP in ACHN and ACHN/RR (in 30 min to 1 h). AR- A014418 and rapamycin combination showed additivity at lower concentrations, but antagonism at higher concentrations.

**Conclusions:**

GSK-3β could directly phosphorylate 4EBP1 and activate the mTORC1 downstream signaling cascades to enhance protein biosynthesis and cell proliferation in RCC cell lines independent of rapamycin sensitivity. The direct GSK-3β/4EBP1 pathway might be an important subcellular mechanism as an inherent equipment for RCC cells to acquire clinical chemoresistance to mTORC1 inhibitors.

**Electronic supplementary material:**

The online version of this article (doi:10.1186/s12885-016-2418-7) contains supplementary material, which is available to authorized users.

## Background

Renal cell carcinoma (RCC) is the most frequent form of kidney cancer, accounting for up to 85 % of all cases [1]. Approximately 25–30 % of patients have metastatic RCC (mRCC) at initial diagnosis [1]. The survival of patients with mRCC has recently prolonged drastically, owing to the development of novel targeted drugs [2]. In most cases, tyrosine kinase inhibitors (TKIs) targeting vascular endothelial growth factor are preferably administered as the first-line drugs for treating mRCC [2, 3]. Two rapalogues that inhibit mammalian target of rapamycin complex 1 (mTORC1), everolimus and temsirolimus, have also been introduced in clinical practice for treating patients with mRCC [2, 3]. However, it is still extremely rare to achieve a complete response with these drugs [2, 4]. Patients with mRCC succumb to the disease once they lapse into refractory status. Thus, further improvement of the therapeutic modality is warranted.

The phosphoinositide 3-kinase (PI3K)/Akt/mTORC1 signaling pathway is an important regulator of cell growth, cell cycling, cell proliferation, metabolism, apoptosis, autophagy, and angiogenesis [5, 6], and is frequently activated in a wide variety of cancers, including RCC [5, 7]. mTORC1 controls these numerous cellular functions mainly via the best-characterized substrates ribosomal protein S6 kinase (S6K) and eukaryotic translation initiation factor 4E (eIF4E)-binding protein 1 (4EBP1) [5, 6]. S6K and 4EBP1 collaborate to play a role in 5′ cap-dependent mRNA translation [6]. S6K stimulates protein synthesis and cell growth, whereas 4EBP1 plays a predominant role in cell proliferation [8]. Experiments involving inhibition of mTORC1 with rapamycin have revealed the differential regulation between its two downstream substrates, S6K and 4EBP1, in a cell type-specific manner [9]. Furthermore, recent reports have suggested that 4EBP1 phosphorylation is directly correlated with the malignancy and severity of various tumors, including RCC [10, 11]. Although mTORC1 seems to be the chief phosphorylation pathway of 4EBP1, other unidentified kinases and biochemical mechanisms are also involved such as cyclin-dependent kinase 1, ataxia-telangiectasia mutated/p53, RAS/extracellular signal-regulated kinase1/2 and other collateral signaling pathways [10, 12]. This is because multiple phosphorylation sites in 4EBP1 can be partially insensitive to rapamycin [10, 12–15]. It is well-known that the mTOR pathway is also an important regulator of hypoxia inducible factor, an essential driver of clear cell RCC due to disruption of the von Hippel Lindau (*VHL*) tumor suppressor gene, the somatic mutation of which is the most frequent genetic alternation observed in RCC [16].

Glycogen synthase kinase-3 (GSK-3) is a ubiquitously expressed Ser/Thr kinase that regulates cellular function via several mechanisms, including Wnt/β-catenin and Hedgehog signal transduction, protein synthesis, glycogen metabolism, mitosis, and apoptosis [17–19]. GSK-3 has two closely related isoforms, GSK-3α and GSK-3β, which exhibit 97 % sequence identity within their catalytic domains [17, 19]. GSK-3 can act as a tumor suppressor or it can promote cell proliferation in different types of cancers [17–19]. We previously demonstrated that GSK-3β positively regulates the proliferation, survival, and anti-apoptosis mechanisms of cancer cells through decreased expression of the nuclear factor-kappa B target genes *BCL-2* and X-linked inhibitor of apoptosis protein (*XIAP*) [18, 20], and that nuclear accumulation of GSK-3β could be a novel biomarker of bladder cancer [18] and RCC [20]. Furthermore, we demonstrated that nuclear overexpression of GSK-3β and tumor proliferation in RCC are negatively regulated by miR-199, the only microRNA known to target GSK-3β [21]. Moreover, pharmacological inhibition of GSK-3 was found to potentiate the anti-tumorous efficacy of sorafenib, a TKI that is used for systemic therapy of mRCC [22].

In the present study, we investigated direct relationships between GSK-3β and 4EBP1 using human RCC cell lines and a normal renal tubular epithelial cell line, and normal renal tissues obtained from RCC patients who had surgical resection, in order to study the role of GSK-3 in the Akt/mTORC1/4EBP1 pathway in RCC.

## Methods

### Cell culture and reagents

The RCC cell lines ACHN, Caki1, and A498 were obtained from American Type Culture Collection (Manassas, VA, USA). ACHN is derived from pleural effusion in metastatic RCC having wild type of *VHL* [23, 24]. Caki1 and A498 cells come from clear cell RCC with *VHL* wild type [23, 25], and clear cell RCC with *VHL* mutation (426_429delTGAC) [25], respectively. Cells were cultured in RPMI medium supplemented with 50 μg/mL of kanamycin and 10 % fetal bovine serum in an incubator at 5 % CO_2_ and 37 °C. Human renal proximal tubular epithelial cell (HRPTEpC) was obtained from Cell applications Inc (San Diego, CA, USA). Cells were cultured in RenaEpi cell growth medium with growth supplements in an incubator at 5 % CO_2_ and 37 °C. AR-A014418 was purchased from Calbiochem (San Diego, CA, USA). Two other GSK-3 inhibitors, SB-216763 and TDZD8, were obtained from Cayman Chemicals (Ann Arbor, MI, USA) and Sigma-Aldrich Japan (Tokyo, Japan), respectively. Rapamycin and everolimus were obtained from Selleck Chemicals (Houston, TX, USA), LY294002 was from Wako Pure Chemical Industries (Tokyo, Japan), recombinant GSK-3β was purchased from New England Biolabs (NEB) Japan (Tokyo, Japan), and recombinant GST-4EBP1 was obtained from Sigma-Aldrich Japan.

### Induction of rapamycin-resistant renal cancer cell lines

The RCC cell line ACHN was cultured in progressively increasing dose of rapamycin until sustained growth, used concentration ranging from 1nM finally to 1 μM (for approximately 4 months). Before use the rapamaycin-resistant cells to investigate drug effects, the cells were cultured in RPMI medium without rapamycin for five passages.

### siRNA transfection

For GSK-3β or GSK-3α silencing, ACHN cells were transfected with specific human siRNAs against GSK3β (25 μM or 50 μM) or GSK3α (50 μM) by using Lipofectamine RNAiMAX (Invitrogen, Thermo Fisher Scientific Inc. Yokohama, Japan) according to the manufacture’s recommendations. Targeting sequences of siRNA are as follows: GSK-3β; 5′-GGACAAGAGAUUUAAGAAUtt-3′(Applied BioSystems, Thermo Fisher Scientific Inc.), GSK-3α (siE523); 5′-GUCCUCACAAGCUUUAACUtt-3′; GSK-3α (siE524); 5′-GUCUUAGUUUCCACAGUAAtt-3′ (TaKaRa Bio Inc., Shiga, Japan). Non-specific control siRNA (Applied BioSystems) was used as negative control.

### Preparation of normal human kidney tissues

Fresh frozen tissue samples obtained from three patients with RCC who underwent nephrectomy at Yamagata University Hospital were used in the present study. The samples cut from the non-tumorous renal parenchyma away from RCC areas were freshly frozen and maintained at −80 °C until the experiments. The study was approved by the Ethics Committee of Yamagata University Faculty of Medicine (approval no. 55, 2015), and all patients signed an informed consent form.

### Immunoblot analysis

Immunoblot analysis was performed as described previously [22], using SuperSignal West Pico Substrate (Pierce, Rockford, IL, USA) and Western BLoT Hyper HRP Substrate (Takara Bio Inc) according to the manufacturers’ instructions. The images were analyzed using UN-SCAN-Itgel Automated Digitizing System software (Version 5.1 for Windows, Silk Scientific Inc., Orem, UT, USA). The antibodies to the following chemicals were used: 4EBP1, p4EBP1 (The70, Thr37/46, and Ser65), S6K, pS6K (Ser371), ribosomal protein S6 (S6RP), pS6RP (Ser240/244), glycogen synthase (GS), pGS (Ser641), Akt, pAkt (Ser473), GSK-3β and GSK-3α. These antibodies were obtained from Cell Signaling Technology Japan (Osaka, Japan). β-actin was used as a loading control and anti-β-actin was obtained from Abcam Inc. (Cambridge, MA, USA).

### Protein kinase assays

Kinase assays were performed for 30 min at 30 °C with 0.5 μL of recombinant GSK-3β (NEB) and 0.5 μg of recombinant GST-4EBP1 in kinase buffer (50 mM Tris–HCl, 50 mM NaCl, 5 mM dithiothreitol, 1 mM ethylenediaminetetraacetic acid (EDTA), 50 % glycerol, and 0.03 % Brij 35, pH 7.5) containing 500 μM ATP in the presence and absence of 25 μM AR-A014418 or SB-216763. The reaction products were subjected to sodium dodecyl sulfate-polyacrylamide gel electrophoresis, and immunoblot analysis was performed.

### Cell proliferation assay

ACHN, Caki1, and A498 RCC cells were cultured at 24, 48, and 72 h in the presence and absence of an mTORC1 or GSK-3 inhibitor. The cell viability was estimated using CellTiter 96 Aqueous One Solution Cell Proliferation Assay (Promega, Madison, WI, USA) as described previously [21]. Values of the half maximal inhibitory concentration (IC_50_) were calculated by fitting concentration-response curves to a four-parameter logistic sigmoidal function model using R package ‘drc’ (http://www.bioassay.dk). Synergistic, additive, or antagonistic effects of AR-A014418 and rapamycin combination treatment were determined based on the theorem of Chou and Talalay [26], using a free software CompuSyn (www.combosyn.com,). The dose–effect relationships for single agents and their combinations were analyzed, and the combination index (CI) values were calculated for each dose and the corresponding effect level, designated as the fraction affected (Fa) meaning the inhibited fraction of cell proliferation after drug administration. For graphical presentation of drug interaction, a Fa-CI plot was constructed by simulating CI values with CompuSym over a range of Fa levels from 0.05 to 0.97. The CI values provide a quantitative definition for an additive effect (CI = 1), synergism (CI < 1), and antagonism (CI > 1) in drug combinations.

### Cell cycle analysis

After treatment with AR-A014418 or rapamycin, ACHN cells were harvested with trypsin-EDTA, centrifuged into a pellet, and rinsed with phosphate-buffered saline (PBS). Then, 80 % ethanol was added, and the cells were incubated on ice for 1 h. The cells were washed with PBS, re-suspended in PBS containing 20 μg/mL RNase and 50 μg/mL propidium iodide (Sigma-Aldrich Japan), and incubated at 37 °C for 1 h. The cells were analyzed using the FACSCanII flow cytometry (Becton-Dickinson, San Diego, CA, USA).

### Statistical analysis

Continuous variables are presented as the mean ± standard deviation (SD) for bar-charts and as the mean ± standard error (SE) for concentration-response plots. They were statistically analyzed using analysis of variance (ANOVA) and, if necessary, a post-hoc Bonferroni test for multiple comparisons. *P* < 0.05 was considered statistically significant. All analyses were performed using R statistical software version 3.1.0 (http://cran.r-project.org/).

## Results

### GSK-3 inhibition shows different modes of anti-proliferative action in ACHN cells

**Fig. 1:**
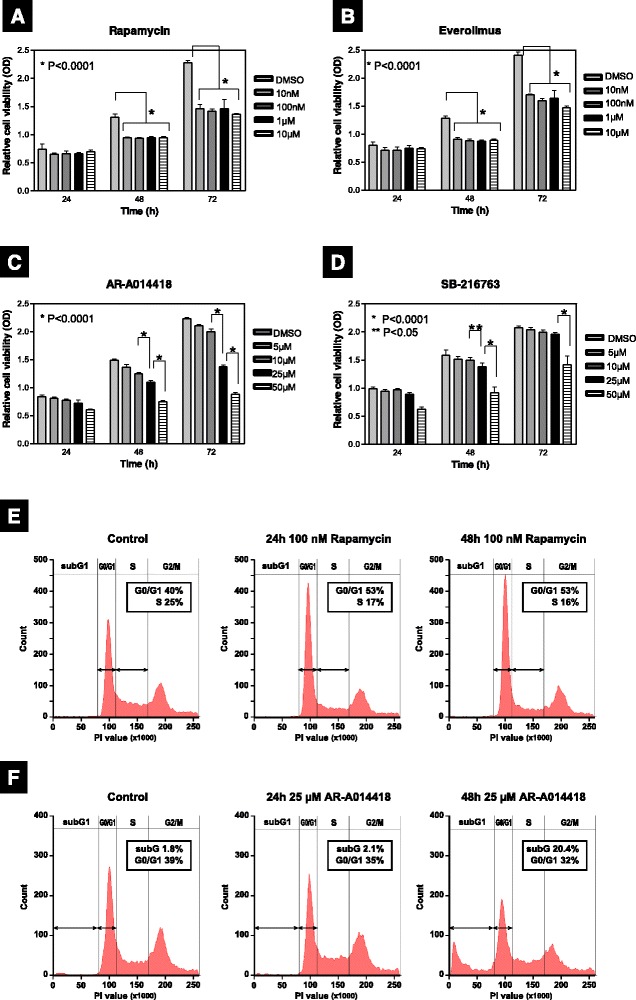
Differences in the suppressive effects of mTORC1 and GSK-3 inhibitors on cell proliferation. **a**–**d** Relative cell viability was measured by an MTS assay in ACHN cells treated with rapamycin (**a**), everolimus (**b**), AR-A014418 (**c**), and SB-216763 (**d**) at the indicated concentrations at 24, 48, and 72 h, respectively. Data are the mean ± SD in each bar-plot from six replicates of each cell line. **e** and **f** Cell cycle distribution in the presence of 100 nM of rapamycin (**e**) or 25 μM AR-A014418 (**f**) at 24 and 48 h. Data are representative of at least three independent experiments

We investigated cell proliferation in ACHN RCC cells treated with inhibitors of mTORC1 or GSK-3. Treatment of rapamycin and its derivative everolimus, both of which are inhibitors of mTORC1, reduced cell viability, and the ACHN cell viability leveled off when the concentrations of the rapalogues were higher than 10 nM (Fig. 1a and b). By contrast, AR-A014418 and SB-216763, both of which are ATP-competitive and small-molecule inhibitors of GSK-3 [27, 28], decreased cell viability in a concentration-dependent manner (Fig. 1c and d). We further investigated the cell proliferation of human RCC cell lines (ACHN, Caki1 and A498) using in detail-concentration of AR-A014418 and rapamycin (Additional file 1: Figure S1). Generally, all the three cell lines were less sensitive to rapamycin than AR-A014418, not having reached 50 % inhibition of cell viability from baseline even at high doses of rapamycin (1000 nM), whereas the cell lines showed a dose-dependent decrease in proliferation with IC_50_ between 27 and 45 μM treated with AR-A014418 (Additional file 1: Figure S1). Rapamycin caused G0/G1 arrest in ACHN cells without inducing apoptosis (Fig. 1e). Similar to our previous study [14], AR-A014418 induced apoptosis in conjunction with the increase in the subG1 phase at 48 h (Fig. 1f). Using immunoblotting, we confirmed that GSK-3 inhibition with AR-A014418 induced dose- and time-dependent apoptosis, as measured by poly ADP ribose polymerase (PARP) cleavage and reduction of XIAP (data not shown). In contrast, AR-A014418 also reduced the G0/G1 phase at 24 h independently of the increase in the subG1 phase, implicating the possibility of anti-mitotic action that is independent of apoptosis (Fig. 1f).

### Pharmacological and genetic inhibition of GSK-3 prevents the downstream phosphorylation of mTORC1

**Fig. 2:**
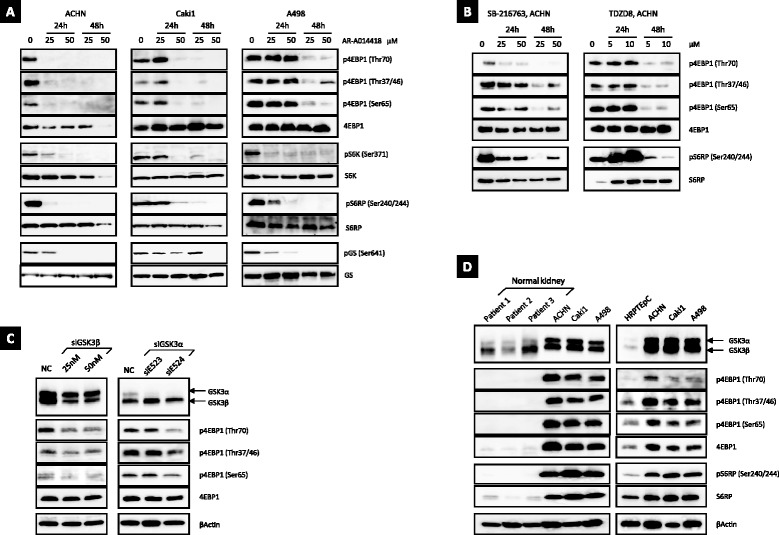
Pharmacological and genetic inhibition of GSK-3 suppresses 4EBP1, S6K, and S6RP phosphorylation in the mTORC1 downstream signaling cascade aberrantly activated in RCC cell lines. **a** The human RCC cell lines ACHN, Caki1, and A498 cells were treated with AR-A014418 (25 μM or 50 μM) for 24 h or 48 h, respectively. **b** ACHN cells were treated with SB-216763 (25 μM or 50 μM), and with TDZD8 (5 μM or 10 μM) for 24 h or 48 h, respectively. **c** ACHN cells were transfected with non-specific control siRNA (50 nM) as negative control (NC), GSK-3β-targeting (siGSK3β) siRNAs (25 nM or 50 nM) and GSK-3α-targeting (siGSK3α) siRNAs (50nM) for 48 h after seeding. **d** Human renal proximal tubular epithelial cell (HRPTEpC) and normal kidney tissues had slight expression of GSK-3 and the mTORC1 downstream substances (p4EBP1, pS6K and pS6RP). In contrast, GSK-3 and the mTORC1 downstream signaling pathway were strongly activated in RCC cells. In **a**–**d**, immunoblot analysis was performed after cell lysis. Data are representative of at least three separate experiments

To examine the role of GSK-3 in the mTORC1 downstream signaling pathway and its effects on cell proliferation, we treated ACHN, Caki1, and A498 human RCC cells, with AR-A014418, an ATP-competitive and small-molecule inhibitor of GSK-3 [27], and examined its effects on the phosphorylation of 4EBP1, S6K, and S6RP (Fig. 2a). Immunoblot analysis showed that p4EBP1 at Thr70, Thr37/46, and Ser65 were suppressed with treatment of AR-A014418 in all the three cell lines. In parallel, AR-A014418 simultaneously inhibited the phosphorylation of S6K at Ser371 and its downstream substrate pS6RP at Ser240/244. Treatment with two other GSK-3 inhibitors, SB-216763 (ATP-competitive) [28] and TDZD8 (ATP-non-competitive) [29], also reduced p4EBP1 and pS6RP levels in ACHN cells (Fig. 2b). GSK-3 has a primary role in the phosphorylation of glycogen synthase (GS), and it is known to directly phosphorylate S6K at Ser371 in vitro. [30] Here, we found that AR-A014418 induced dose- and time-dependent inhibition of GSK-3 activity, as measured by pGS at Ser641 and pS6K at Ser371 levels (Fig. 2a).

Next, to determine which of two GSK-3 isoforms, GSK-3α and GSK-3β, phosphorylates 4EBP1, we examined p4EBP1 status in stable GSK-3α and GSK-3β knockdown cells with siRNAs, respectively (Fig. 2c). As expected, 4EBP1 phosphorylation at Thr70, Thr37/46 and Ser65 in siGSK-3β-transfected ACHN cells decreased than negative controls. However, siGSK-3α knockdown presented with inconsistent outcome, no changes and reduction in p4EBP1 for each of two specific siGSK-3α transfected, respectively (Fig. 2c).

Compared with the human renal proximal tubular epithelial cells (HRPTEpC) and the normal renal parenchymas, the mTORC1 downstream pathway was strongly activated in the RCC cell lines (Fig. 2d). These results indicate that GSK-3β, rather than GSK-3α, is required to maintain 4EBP1, S6K, and S6RP phosphorylation in RCC cells.

### GSK-3 regulates 4EBP1 and S6RP phosphorylation differently from PI3K/Akt/mTORC1 pathway

**Fig. 3:**
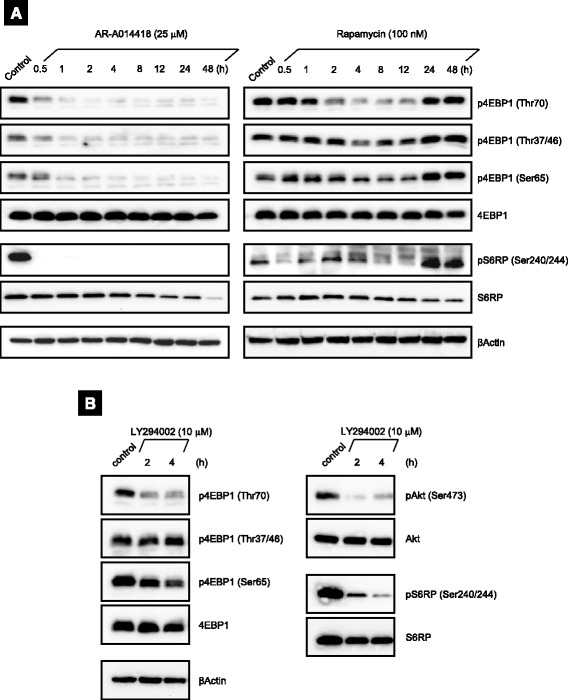
GSK-3 differentially regulates 4EBP1 and S6RP phosphorylation in ACHN cells. **a** Changes in the phosphorylation levels of 4EBP1 and S6RP over time after treatment of AR-A014418 (25 μM) or rapamycin (100 nM) to ACHN cells. **b** ACHN cells were treated with LY294002 (10 μM) for the indicated times. In **a** and **b**, the cells were lysed and then analyzed for the phosphorylation of 4EBP1 and S6RP by immunoblotting. Data are representative of at least three independent immunoblot experiments

To investigate the relationship GSK-3 and mTORC1 pathway further, we next examined the effect of AR-A014418 and rapamycin on phosphorylation of 4EBP1 and S6RP, the downstream signaling targets of mTORC1. The phosphorylation levels of 4EBP1 and S6RP in ACHN cells decreased rapidly at 30 min after exposure to AR-A014418, and the phosphorylation remained suppressed through 48 h (Fig. 3a, *left*). In contrast, rapamycin temporarily suppressed 4EBP1 phosphorylation at Thr70 from 2 to 12 h and at Thr37/46 and Ser65 from 4 to 12 h, but did not prevent phosphorylation at Thr37/46, Thr70 and Ser65 at 24 and 48 h. S6RP phosphorylation was partially suppressed from 0.5 to 12 h, and were finally restored at 24 and 48 h as well as p4EBP1 (Fig. 3a, *right*). Similar to rapamycin, LY294002, a PI3K/Akt/mTORC1 inhibitor, moderately attenuated p4EBP1 at Thr70 and Ser65 without apparent changes at Thr37/46 phosphorylation. As expectedly, LY294002 also inhibited pAkt and pS6RP simultaneously (Fig. 3b). To our investigation, the effect of AR-A014418 on Akt phosphorylation at Ser473 varied according to the concentrations and RCC cell lines (Additional file 2: Figure S2). However, p4EBP1 commonly decreased in ACHN, Caki1, and A498 cells following AR-A014418 treatment (Fig. 3a and Additional file 2: Figure S2).

These results support that GSK-3 regulates the phosphorylation of 4EBP1 directly and S6K differentially in a manner independent of the PI3K/Akt/mTORC1 pathway. In addition, it reflects that 4EBP1 would take phosphorylation differentially at Thr70, Thr37/46 and Ser65.

### GSK-3β could directly phosphorylate 4EBP1

**Fig. 4:**
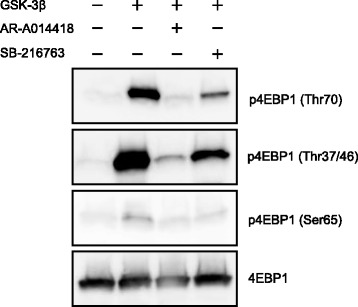
In vitro kinase assays. In vitro kinase assays were performed using recombinant GSK-3β and recombinant 4EBP1 as described in the Materials and methods. Either AR-A014418 (25 μM) or SB-216763 (25 μM) was applied in vitro to inhibit recombinant GSK-3β. p4EBP1 levels were measured with immunoblot analysis. Data are representative of three separate immunoblot analysis

We performed an in vitro kinase assay using recombinant proteins of GSK-3β and 4EBP1 (Fig. 4). Recombinant GSK-3β phosphorylated recombinant 4EBP1 at Thr70, Thr37/46, and Ser65. AR-A014418 and SB-216763 blocked the recombinant GSK-3β mediated phosphorylation of recombinant 4EBP1. These results suggest that GSK-3β could participate in the direct phosphorylation of 4EBP1 at Thr70, Thr37/46, and Ser65, as reported for S6K [30].

### Biphasic response to drug combination of GSK-3 and mTORC1 inhibitors in a concentration-dependent fashion

**Fig. 5:**
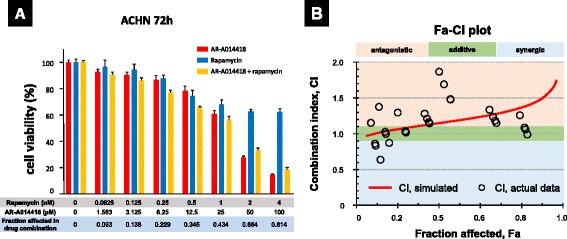
Combination effects of AR-A014418 and rapamycin in ACHN cells. **a** ACHN cells were treated with AR-A014418 and rapamycin as single agents or their combination at different concentration at a constant dilution ratio of 25,000 : 1. Cell viability was assessed using MTS assay after 72 h incubation. Data are the mean ± SD in each bar-plot from four replicates of ACHN cells at each concentration. **b** Effects of the drug combination was demonstrated on a Fa-CI plot generated using CompuSyn. Combination index (CI) determines the type of drug interaction as follows: additive, synergistic, or antagonistic if CI is 1, < 1 or > 1, respectively [26]. The combination effects varied from additivity to antagonism according to the increase in drug concentration

In ACHN cells, we investigated cell proliferation with single agents and a combination of GSK-3 inhibitor (AR-A014418) and mTORC1 inhibitor (rapamycin) using the MTS assay. Different concentration in the combination was kept at a constant dilution ratio of 25,000 : 1. The dilution ratio was determined on IC_50_ values from concentration-response relation in MTS assay at 72 h for the single agents (Fig. 5a) [26]. The combination effects were determined as the combination index with CompuSyn software (Fig. 5b) [26]. The combination effects were additive at lower concentrations, but more antagonistic at higher concentrations.

### GSK-3 regulates cell proliferation and p4EBP1 independently of chemoresistance to rapamycin

**Fig. 6:**
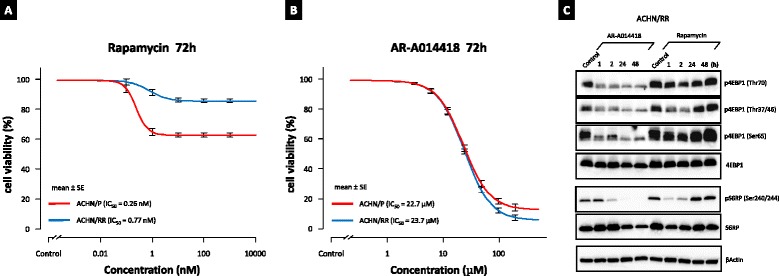
GSK-3 inhibition overcomes rapamycin-resistance in ACHN cells. ACHN/P (parental) and ACHN/RR (rapamycin-resistant) cells were treated with increasing doses of rapamycin (**a**) and AR-A014418 (**b**) at 72 h. Cell viability was measured using MTS assay. Data are the mean ± SE in each from six replicates of each cells. In **a** and **b**, significant interaction between the main effects of cell lines (ACHN/P or ACHN/RR) and drug concentrations was statistically detected with two-way ANOVA (*p* < 0.05). As for the simple main effect, the difference in cell lines (ACHN/P or ACHN/RR) was statistically significant for rapamycin and not significant for AR-A014418 (one-way ANOVA with Bonferroni test; *p* < 0.05 and = 0.71, respectively). **c** ACHN/RR cells were treated with AR-A014418 (25 μM) or rapamycin (100 nM) at indicated times. The cells were lysed and analyzed for the phosphorylation of 4EBP1 and S6RP by immunoblotting. Data are representative of at least three separate immunoblot experiments

To confirm that GSK-3 regulates cell proliferation and 4EBP1 phosphorylation independently of PI3K/Akt/mTORC1 pathway, we furthermore investigated inhibitory effects of AR-A014418 in rapamycin-resistant ACHN (ACHN/RR) cells that were generated through culturing ACHN parental (ACHN/P) cells in progressively higher concentrations of rapamycin from 1nM finally to 1 μM. As shown in Fig. 6a, ACHN/P cells were sensitive to rapamycin in proliferation, but ACHN/RR cells presented with rapamycin-resistance. In contrast, AR-A014418 suppressed greatly ACHN/P and ACHN/RR proliferation in concentration-dependent manner. Notably, the concentration-response relation for AR-A014418 did not differ from ACHN/P and ACHN/RR cells (Fig. 6b). In ACHN/RR cells, AR-A014418 remarkably inhibited p4EBP1 and pS6RP rapidly and the inhibition had continued constantly even at 48 h. However, phosphorylations of 4EBP1 and S6RP in ACHN/RR cells were suppressed only mildly and transiently by rapamycin. The phosphorylations almost recovered to the baseline in 24 h (Fig. 6c). The findings indicate that GSK-3 inhibition can sufficiently decrease the activities of mTORC1 downstream cascades and overcome rapamycin-resistance in RCC cells.

## Discussion

Consistent with previous reports [30, 31], in the present study, we demonstrate that GSK-3β directly phosphorylates 4EBP1 independent of rapamycin sensitivity to mTORC1 and continuously activates 4EBP1 and S6K, the mTORC1 downstream substrates to drive cell proliferation in RCC cell lines. The present findings would also indicate that the direct GSK-3β/4EBP1 pathway is an important subcellular mechanism as an inherent equipment for RCC cells to acquire clinical chemoresistance to mTORC1 inhibitors. Although rapamycin increased the G0/G1 phase in cell cycle and suppressed cell proliferation, the inhibitory effects of rapamycin on the mTORC1 downstream pathway were only transient leading to gradual return of p4EBP1 and pS6RP to the baseline level. When the present results extrapolated into clinical setting, it could be easy for us to understand limited efficacy of mTORC1 inhibition on size reduction and progression of RCC [2, 3]. Our data suggest that GSK-3 inhibition could be a promising strategy for the treatment of mRCC.

GSK-3 in solid cancers have two opposite functions in different types of tumor cells, a suppressor (e.g., some skin and breast cancers) or promoter (e.g., colon, liver, ovarian and pancreatic tumors) [17–19, 32]. However, the underlying mechanisms to regulate the differentiation between the opposite roles of GSK-3 remain unsolved [32]. To date, we have reported that GSK-3β is highly expressed in tumor nuclei of RCC [21]. In human RCC specimens, aberrant GSK-3β overexpression that is negatively regulated by miR-199 [21] was observed in 68 out of 74 (92 %) cases, suggesting clinical relevance of RCC biology [20]. Using RCC cell lines, we have revealed that inhibition of GSK-3 results in decreased expression of NF-kB target genes Bcl-2 and XIAP leading to a subsequent increase in RCC apoptosis and the anti-tumor effect of sorafenib, TKI available for treatment of mRCC in clinical practice [20, 22]. According to a recent review [32], possible differences in apoptotic, Wnt/β-catenin, and NF-kB signaling pathways among various types of cancer may be related to the opposite effects of GSK-3 on tumor proliferation.

Drug combination of AR-A014418 and rapamycin in MTS assay for ACHN proliferation presented with additivity at lower concentrations, but antagonism at higher concentrations. The biphasic aspect, including the transition from additive to antagonistic, with increasing drug concentration indicated the presence of at least two regulatory pathways from GSK-3 to 4EBP1. The additive effect is considered to reflect a direct pathway independent of the PI3K/Akt/mTORC1 cascade. The antagonism at higher concentrations could possibly be competitive inhibition because GSK-3 and mTORC1 in combination share 4EBP1 and S6K as target substrates (Figs. 2, 3 and 6c).

4EBP1 plays an important role in cell proliferation by selectively translating mRNAs that encode various proteins promoting cell cycle and proliferation [8]. Phosphorylation, mainly at sites Thr37, Thr46, Ser65, and Thr70, causes 4EBP1 to dissociate from eIF4E, allowing for cap-dependent translation [33, 34]. It is widely accepted that mTORC1 is a main regulator of 4EBP1 to keep cellular homeostasis [35]. However, as shown in Fig. 2d, 4EBP1 and S6RP expression and phosphorylation aberrantly increased in RCC cells but not in normal kidney tissues and a renal tubular cell line, suggesting that GSK-3 as well as mTORC1 could be involved in the activation of mTORC1 downstream pathway in RCC. Moreover, rapamycin and LY294002 only partially inhibited the phosphorylation of 4EBP1 (Fig. 3a and b) [15]. Not only pharmacological but also genetic inhibition of GSK-3β with specific siRNAs sufficiently decreased phosphorylation of mTORC1 downstream substrates in RCC cell lines (Figs. 1 and 2). Therefore, we here provide the evidence that GSK-3β can directly phosphorylate 4EBP1 at Thr37/46, Thr70, and Ser65, possibly in sequential order (Fig. 4). In particular, p4EBP1 was rapidly inhibited following AR-A014418 treatment at 30 min in ACHN cells (Fig. 3a) and within 1 h in acquired rapamycin-resistant ACHN cells (Fig. 6c), supporting the direct involvement of GSK-3β in 4EBP1 phosphorylation in RCC cells.

Approximately 100 substrates of GSK-3 have been identified to date [17, 19]. Recently, several researchers reported about relationships between GSK-3β and Akt/mTORC1/4EBP1 pathway [31, 36–38]. However, to our knowledge, only two papers have referred to direct interaction of GSK-3β and 4EBP1 currently [31, 38]. According to Shin et al. [31], GSK-3β directly phosphorylates 4EBP1 at Thr37/46 and inactivates 4EBP1 activity in breast cancer and normal cell lines, thereby increasing eIF4E-dependent protein synthesis and regulating cell proliferation. In addition, they showed that GSK-3β significantly reduced in vivo the size of tumor injected subcutaneously in a mouse xenograft model [31].

Many GSK-3β substrates have the motif Ser/Thr-Pro-X-X-pSer/Thr [39]. The binding affinity of GSK-3β to a substrate is enhanced if the substrate receives prior phosphorylation at the second Ser/Thr [19]. 4EBP1 reportedly has seven phosphorylation sites (Thr37/46, Ser65, Thr70, Ser83, Ser101, and Ser112) [14, 39]. Comparison of the 4EBP1 sequence with those of known GSK-3β substrates showed that the best-matched sequences in 4EBP1 for phosphorylation by GSK-3β are Thr-Pro-Gly-Gly-Thr for both Thr37 and Thr46 as the first Ser/Thr residues [40]. Phosphorylation of 4EBP1 at Thr37/46 by GSK-3β requires pre-phoshorylation at priming sites (Thr41/50) [31]. However, GSK-3 does not necessarily require a primed substrate [17, 19, 41]. The residues other than Thr37/46, Thr70, and Ser65 in 4EBP1 might be also targeted by GSK-3β. In the present study, we demonstrated that recombinant GSK-3β phosphorylated an unprimed recombinant 4EBP1 at Thr37/46. It is possible that phosphorylation of Thr41/50 also is regulated by GSK-3β itself. There are no priming phosphorylation sites at Thr70 and Ser65 of 4EBP1 (Thr^70^-Pro-Pro-Arg-Asp, Ser^65^-Pro-Val-Thr-Lys). The phosphorylation of 4EBP1 appears to occur in a hierarchical manner, as follows [13, 14]: Thr37/46 are phosphorylated at baseline, and this is further enhanced by insulin production [13, 14]. Thr37/46 phosphorylation is followed by phosphorylation of Thr70, followed by phosphorylation of Ser65 as the last step [13]. Phosphorylation of Thr70 seems to play the most important role in releasing 4EBP1 from eIF4E [14]. The fact that the phosphorylation levels of recombinant unprimed 4EBP1 increased in the order of Thr37/46, Thr70, and Ser65 likely reflects this hierarchical phosphorylation process (Fig. 4). As shown in Fig. 3, LY294002 and rapamycin exhibited apparently no and slightly inhibitory action on Thr37/46 phosphorylation in 4EBP1, respectively, leading to the notion that Thr37/46 residue may be the main target of GSK-3 in case of 4EBP1 phosphorylation [31]. Abnormal overexpression of GSK-3 is observed in cancers which are resistant to chemo-, radio- and targeted therapy [32]. Targeting GSK-3 could improve cancer therapy and overcome therapeutic resistance [32].

The mechanism by which GSK-3 inhibition results in fluctuations of pAkt at Ser437 over time is currently unclear (Additional file 2: Figure S2). One potential mechanism is that at the early phase, inhibition of GSK-3β by AR-A014418 results in the hypo-phosphorylation of Ser1235 of rictor, a component of mTORC2 that activates Akt via pSer437 [42]. Another possibility is that S6K could participate in negative feedback regulation to affect the phosphorylation of rictor at Thr1135 through the insulin receptor substrate 1/PI3K axis [6, 43]. A third potential mechanism could involve apoptosis-induced protein degradation and the consequent reduction of pAkt [5, 6]. Although GSK-3 inhibition appears to induce variable reactions of pAkt at Ser473 with unknown mechanisms, it consistently suppressed 4EBP1 and mTORC1 downstream signaling in RCC cells in the present study. In their report, Armengol et al. [10] stated that 4EBP1 would act as a key molecular funnel factor in carcinogenesis of various types of human cancers, regardless of the upstream oncogenic alterations. The results of the present study on RCC cells seem to support this hypothesis.

## Conclusions

GSK-3β could directly phosphorylate 4EBP1 and activate the mTORC1 downstream signaling cascades to enhance protein biosynthesis and cell proliferation in RCC cell lines independent of rapamycin sensitivity. The direct GSK-3β/4EBP1 pathway, not associated with the PI3K/Akt/mTORC1 pathway, would be an important subcellular intrinsic mechanism for RCC cells to resist mTORC1 inhibition, possibly leading to potential therapeutic limitations in systemic treatment for mRCC patients with rapalogues.

## Abbreviations

4EBP1, eukaryotic translation initiation factor 4E (eIF4E) -binding protein 1; CI, combination index; eIF4E, eukaryotic translation initiation factor 4E; GS, glycogen synthase; GSK-3, glycogen synthase kinase-3; GST, glutathione S-transferase; mRCC, metastatic renal cell carcinoma; mTOR, mammalian target of rapamycin; mTORC, mammalian target of rapamycin (mTOR) complex; PI3K, phosphoinositide 3-kinase; RCC, renal cell carcinoma; S6K, ribosomal protein S6 kinase; S6RP, ribosomal protein S6; TKI, tyrosine kinase inhibitor; VHL, von Hippel Lindau; XIAP, X-linked inhibitor of apoptosis protein

## Additional files

Additional file 1: Figure S1. Differences in suppressive effects of mTORC1 and GSK-3 inhibitors on cell proliferation. Relative cell viability was measured by an MTS assay in ACHN, Caki1 and A498 cells treated with rapamycin (A), and AR-A014418 (B) at the indicated concentrations at 72 h. A498 cells, highly insensitive to rapamycin, did not reach maximal inhibitory effect even at 10 μM rapamycin, with IC_50_ not assessable (NA). Data are the mean ± SE from six replicates of each cell line. In (A) and (B), significant interaction between cell lines and drug concentrations was statistically detected with two-way ANOVA (*p* < 0.05). As for the simple main effect, the difference in cell lines (ACHN, Caki1 or A498) was statistically significant for rapamycin (one-way ANOVA; *p* < 0.05, Bonferroni test; *p* < 0.05 for ACHN vs. Caki1 and A498) and not significant for AR-A014418 (one-way ANOVA; *p* = 0.47). (PPTX 60 kb) Additional file 2: Figure S2. Different effects of GSK-3 inhibition on Akt and 4EBP1 phosphorylation. (A) ACHN cells were treated with AR-A014418 (25 μM) or rapamycin (100 nM) for the indicated times. Cells were lysed and analyzed for pAkt by immunoblotting. (B) Caki1 and A498 cells were treated with AR-A014418 at 25 μM or 50 μM for 24 h or 48 h, respectively. Immunoblotting was performed after cell lysis. In (A) and (B), Data are representative of at least three separate immunoblot analyses. (PPTX 107 kb)
