# Supplementary material for: GSK-3 directly regulates phospho-4EBP1 in renal cell carcinoma cell-line: an intrinsic subcellular mechanism for resistance to mTORC1 inhibition
Source: BMC Cancer. 2016 Jul 7;16:393. doi: 10.1186/s12885-016-2418-7 (PMC4936323; doi:10.1186/s12885-016-2418-7)
Supplement: Additional file 1: Figure S1. — Differences in suppressive effects of mTORC1 and GSK-3 inhibitors on cell proliferation. Relative cell viability was measured by an MTS assay in ACHN, Caki1 and A498 cells treated with rapamycin (A), and AR-A014418 (B) at the indicated concentrations at 72 h. A498 cells, highly insensitive to rapamycin, did not reach maximal inhibitory effect even at 10 μM rapamycin, with IC50 not assessable (NA). Data are the mean ± SE from six replicates of each cell line. In (A) and (B), significant interaction between cell lines and drug concentrations was statistically detected with two-way ANOVA (p < 0.05). As for the simple main effect, the difference in cell lines (ACHN, Caki1 or A498) was statistically significant for rapamycin (one-way ANOVA; p < 0.05, Bonferroni test; p < 0.05 for ACHN vs. Caki1 and A498) and not significant for AR-A014418 (one-way ANOVA; p = 0.47). (PPTX 60 kb) [file 12885_2016_2418_MOESM1_ESM.pptx]

## Slide 1
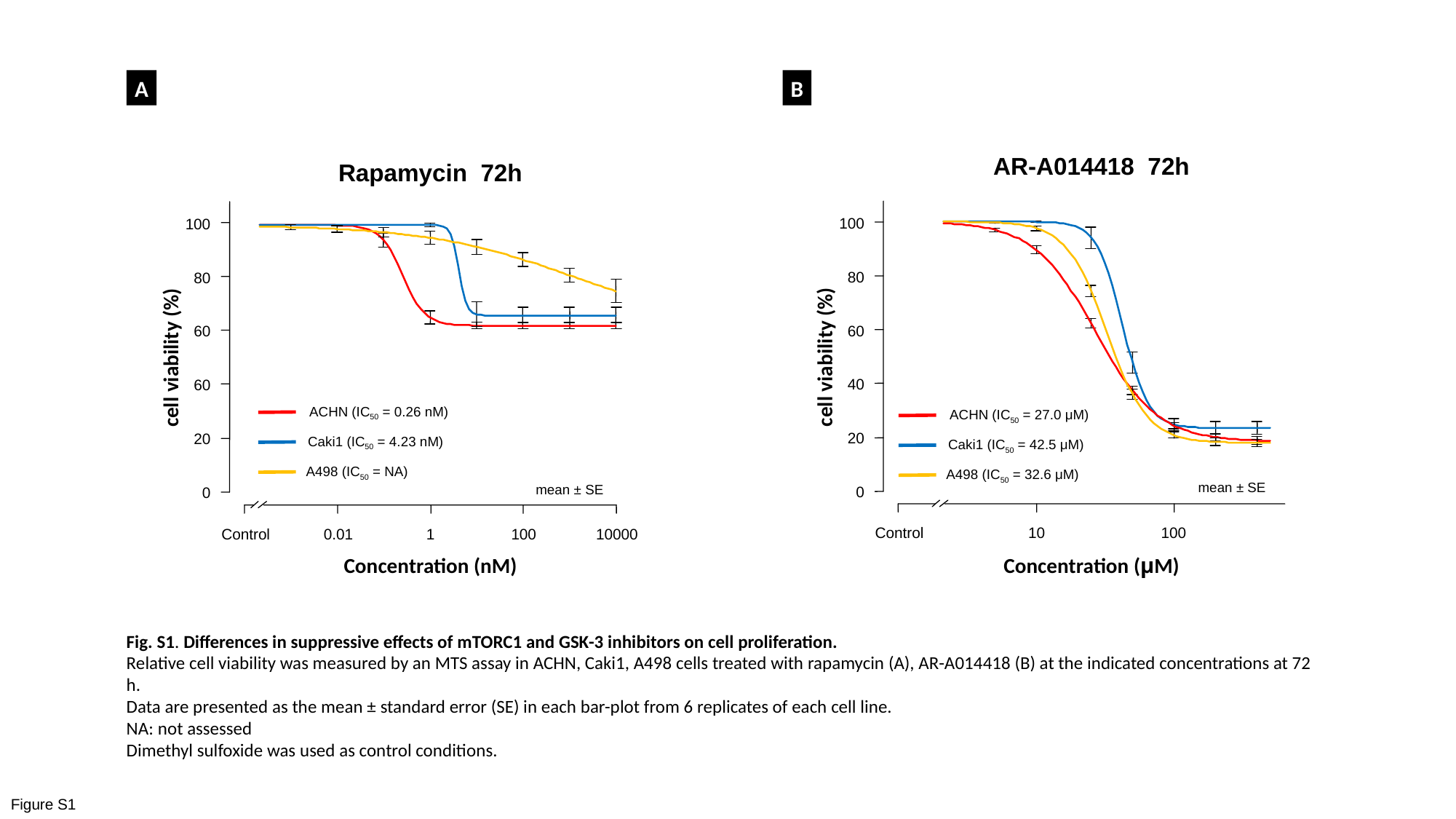

A
B
AR-A014418 72h
100
80
60
40
20
0
cell viability (%)
ACHN (IC50 = 27.0 μM)
Caki1 (IC50 = 42.5 μM)
A498 (IC50 = 32.6 μM)
mean ± SE
Control
10
100
Concentration (μM)
Rapamycin 72h
100
80
60
60
20
0
cell viability (%)
ACHN (IC50 = 0.26 nM)
Caki1 (IC50 = 4.23 nM)
A498 (IC50 = NA)
Control
0.01
1
100
10000
Concentration (nM)
mean ± SE
Fig. S1. Differences in suppressive effects of mTORC1 and GSK-3 inhibitors on cell proliferation.
Relative cell viability was measured by an MTS assay in ACHN, Caki1, A498 cells treated with rapamycin (A), AR-A014418 (B) at the indicated concentrations at 72 h.
Data are presented as the mean ± standard error (SE) in each bar-plot from 6 replicates of each cell line.
NA: not assessed
Dimethyl sulfoxide was used as control conditions.
Figure S1
