# Supplementary material for: GSK-3 directly regulates phospho-4EBP1 in renal cell carcinoma cell-line: an intrinsic subcellular mechanism for resistance to mTORC1 inhibition
Source: BMC Cancer. 2016 Jul 7;16:393. doi: 10.1186/s12885-016-2418-7 (PMC4936323; doi:10.1186/s12885-016-2418-7)
Supplement: Additional file 2: Figure S2. — Different effects of GSK-3 inhibition on Akt and 4EBP1 phosphorylation. (A) ACHN cells were treated with AR-A014418 (25 μM) or rapamycin (100 nM) for the indicated times. Cells were lysed and analyzed for pAkt by immunoblotting. (B) Caki1 and A498 cells were treated with AR-A014418 at 25 μM or 50 μM for 24 h or 48 h, respectively. Immunoblotting was performed after cell lysis. In (A) and (B), Data are representative of at least three separate immunoblot analyses. (PPTX 107 kb) [file 12885_2016_2418_MOESM2_ESM.pptx]

## Slide 1
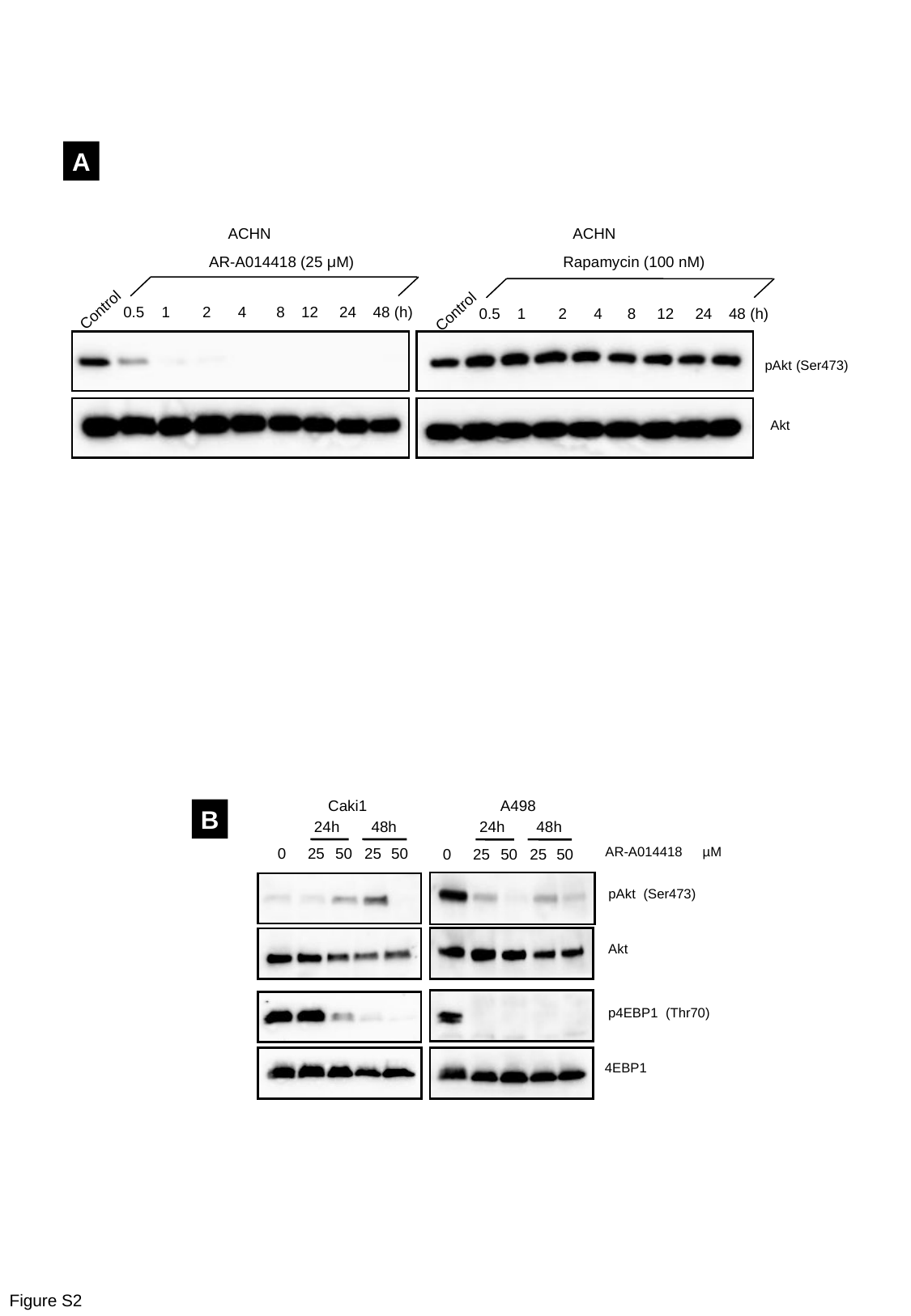

A
ACHN
ACHN
AR-A014418 (25 µM)
Rapamycin (100 nM)
Control
Control
0.5
1
2
4
8
12
24
48 (h)
0.5
1
2
4
8
12
24
48 (h)
pAkt (Ser473)
Akt
Caki1
A498
B
24h
48h
24h
48h
AR-A014418　µM
0
25
50
25
50
0
25
50
25
50
pAkt (Ser473)
Akt
p4EBP1 (Thr70)
4EBP1
Figure S2
